# Comparing indices of responsiveness for the Coma Near‐Coma Scale with and without pain items: An Exploratory study

**DOI:** 10.1002/brb3.3120

**Published:** 2023-06-11

**Authors:** Jennifer A. Weaver, Vera Pertsovskaya, Jasmine Tran, Allan J. Kozlowski, Ann Guernon, Theresa Bender Pape, Trudy Mallinson

**Affiliations:** ^1^ Department of Occupational Therapy, College of Health and Human Sciences Colorado State University Fort Collins Colorado; ^2^ Department of Clinical Research and Leadership, School of Medicine and Health Sciences The George Washington University Washington District of Columbia; ^3^ Mary Free Bed Rehabilitation Hospital Grand Rapids Michigan; ^4^ Speech‐Language Pathology Program, College of Nursing and Health Sciences Lewis University Romeoville Illinois; ^5^ Neuroplasticity in Neurorehabilitation Lab Hines Veterans Affairs Hospital Hines Illinois; ^6^ Department of Physical Medicine and Rehabilitation Northwestern University, Feinberg School of Medicine Chicago Illinois

**Keywords:** brain injuries, consciousness disorders, pain, rehabilitation

## Abstract

**Introduction:**

This study aimed to establish the indices of responsiveness for the Coma/Near‐Coma (CNC) scale without (8 items) and with (10 items) pain test stimuli. A secondary purpose was to examine whether the CNC 8 items and 10 items differ when detecting change in neurobehavioral function.

**Methods:**

We analyzed CNC data from three studies of participants with disorders of consciousness: one observational study and two intervention studies. We generated Rasch person measures using the CNC 8 items and CNC 10 items for each participant at two time points 14 ± 2 days apart using Rasch Measurement Theory. We calculated the distribution‐based minimal clinically important difference (MCID) and minimal detectable change using 95% confidence intervals (MDC_95_).

**Results:**

We used the Rasch transformed equal‐interval scale person measures in logits. For the CNC 8 items: Distribution‐based MCID 0.33 SD = 0.41 logits and MDC_95_ = 1.25 logits. For the CNC 10 items: Distribution‐based MCID 0.33 SD = 0.37 logits and MDC_95_ = 1.03 logits. Twelve and 13 participants made a change beyond measurement error (MDC_95_) using the CNC 8‐item and 10‐item scales, respectively.

**Conclusion:**

Our preliminary evidence supports the clinical and research utility of the CNC 8‐item scale for measuring the responsiveness of neurobehavioral function, and that it demonstrates comparable responsiveness to the CNC 10‐item scale without administering the two pain items. The distribution‐based MCID can be used to evaluate group‐level changes while the MDC_95_ can support clinical, data‐driven decisions about an individual patient.

## Graphical abstract

Our article identifies the indices of responsiveness for the Coma Near Coma Scale, with and without pain items. When using the Coma Near Coma‐8 item or 10‐item, a change score of 1.25 or 1.03 is needed, respectively, to know whether the patients progress or decline is beyond measurement error and reflects true change. Our results substantiates that the 8‐item Coma Near Coma scale has comparable reliability and precision to detect change compared to the 10‐item version.

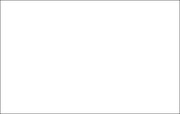

Clinicians and researchers rely on standardized assessment data to interpret whether patients in disorders of consciousness are making a change in neurobehavioral function (i.e., recovery of consciousness). Evidence of psychometric properties for standardized, bedside observational assessments has grown in recent years (Pape et al., 2014; Weaver et al., 2022; 2020; Williams & Smith, 2017). A systematic review of assessments for disorders of consciousness identified there is a need for reliable and valid assessments to examine a critical gap in measurement, the assessment's ability to detect change (Seel et al., 2010). Only one neurobehavioral function assessment, the Disorders of Consciousness Scale‐25, has quantified meaningful change by reporting indices of responsiveness (Mallinson et al., 2016).

Indices of neurobehavioral responsiveness can include the anchor‐based and distribution‐based minimal clinically important difference (MCID) and minimal detectable change based on a 95% confidence interval (MDC_95_). Anchor‐based MCIDs map patient change on an assessment to an external criterion that is considered important to family, care partners, patients, or clinicians (Crosby et al., 2003; Guyatt et al., 2002). To the best of our knowledge, there is currently no assessment of neurobehavioral function for which care partners’ or clinicians’ perceptions of change is the external criterion for an anchor‐based MCID. The distribution‐based MCID indicates whether the difference between scores from two time points are likely to represent a group‐level change that exceeds a clinical standard. The distribution‐based MCID represents the smallest amount of change in an outcome that might be considered important and provides an estimate of treatment effectiveness in research. The MDC_95_ indicates whether the difference between scores from two time points are likely to represent an individual‐level change that exceeds measurement error. Establishing indices of responsiveness, such as the MDC_95_, is important because clinicians must monitor neurobehavioral recovery of consciousness using validated measures to determine whether rehabilitation treatment is effective (Giacino et al., 2020). Furthermore, measuring a decline, that is beyond measurement error, in neurobehavioral recovery may signal the need to determine the presence of a new or previously undetected debilitating condition (e.g., subclinical seizures or hydrocephalus).

Assessments evaluating the neurobehavioral function for patients with disorders of consciousness often include items with painful stimuli (e.g., Coma/Near‐Coma Scale, Coma Recovery Scale‐Revised, and Disability Rating Scale) (Zasler et al., 2022). Previous work has identified the need to assess nociception in patients with disorders of consciousness (Schnakers et al., 2010). While localization to noxious stimulation is considered indicative of conscious perception (Giacino et al., 2002), painful stimuli has produced brain activation in the primary somatosensory cortex of patients in a vegetative state (Laureys et al., 2002). Thus, response to painful stimuli occurs when individuals are in the vegetative state, minimally conscious state, and emerged. Identification of nociception may support clinical decisions to adjust the individual's positioning or provide pain medication to increase the comfort of the individual but may not clearly distinguish different levels of consciousness. Prior research on the Coma/Near‐Coma (CNC) scale found that responses to painful stimuli and neurobehavioral function are two distinct concepts (Weaver et al., 2020). This earlier work indicates the CNC scale is a unidimensional assessment of neurobehavioral function only when painful test stimuli/items are not included. This finding suggests that when responses to painful stimuli change over time, the changes do not necessarily reflect changes in neurobehavioral function. To advance our understanding of this finding, the purpose of this study is to (i) compute the indices of responsiveness for the CNC scale without (8 items) and with (10 items) painful test stimuli, and (ii) determine whether the CNC 8 items and CNC 10 items differ according to detection of change in neurobehavioral function.

## 1 METHODS

### 1.1 Participants

This retrospective cohort study includes 40 adults with severe brain injury. Data were included from three studies: (1) Post‐Acute Care Study (*n* = 18), an observational study of patients receiving rehabilitation services (Pape et al., 2014); (2) Familiar Auditory Sensory Training (*n* = 14), a clinical trial examining neurobehavioral function when provided a familiar voice intervention (Bender Pape et al., 2015); and (3) repetitive transcranial magnetic stimulation study (*n* = 8), a clinical trial examining neurobehavioral function in response to a neuromodulatory intervention (Pape, 2015; Zilliox et al., 2022). During study enrollment, participants’ demographics and medical history were obtained through review of health records and interviews with family. For each of the three studies, participants were evaluated weekly after study enrollment until recovery of consciousness (Bender Pape et al., 2015; Pape, 2015; Pape et al., 2014; Zilliox et al., 2022). Ethical approval for this secondary data analysis was obtained from the Institutional Review Board at George Washington University. Across studies, eligibility criteria were: (1) ≥ 18 years, (2) diagnosed with disorders of consciousness from brain injury, and (3) had two CNC scale assessments 14 ± 2 days apart. Participants were excluded from the study if brain injury was due to cancer, tumor, or encephalopathy.

### 1.2 Coma/Near‐Coma Scale

The CNC scale is a short assessment (11 items) originally designed to capture neurobehavioral responses in persons in lower states of disorders of consciousness (Rappaport, 2005, 1992). The assessment scoring form and training recommendations can be found on the Traumatic Brain Injury Model Systems website (Rappaport, 2000). The olfactory item was not administered in any of the three studies due to difficulty in controlling for consistency and shipping restrictions for ammonia; thus, the scale was administered with 10 items (Bender Pape et al., 2015; Pape, 2015; Pape et al., 2014; Zilliox et al., 2022). Each item is scored using a 3‐point rating scale with response options of 0, 2, 4; a lower score indicates better neurobehavioral function (Rappaport, 2000). For example, for the Auditory item a “0” reflects that the patient responds to the bell ringing stimulus with behaviors such as eye opening or orientation toward sound and that this occurs ≥ 3 times. Whereas, for the Vocalization item a “0” reflects spontaneous words (Rappaport, 2000).

The study reported here compares the CNC scale comprised of the 10 items (excluding the olfactory item) and the CNC scale comprised of 8 items (excludes two pain items) (Weaver et al., 2020). For our analyses, the 3‐point rating scale was rescored to 2, 1, 0, so that a higher score indicates better neurobehavioral function. Previous work, identified that the Wright's Person Separation Reliability was 0.87 and 0.89 for the CNC 8 items and CNC 10 items, respectively, indicating the CNC scale is sufficiently precise for group‐level decisions (Weaver et al., 2020). These person separation reliability values are close to the 0.90 threshold for making consistently reliable individual decisions (Kerlinger & Lee, 2000; Van de Winckel A et al., 2022).

### 1.3 Data analyses

For the CNC 8 items and CNC 10 items, we confirmed the Wright's Person Separation Reliability using Winsteps software version 5.3.3.1, because our study includes eight participants that were not in the previous CNC scale psychometric analyses (Weaver et al., 2021). We hypothesized the person separation reliability coefficients would remain the same because a strength of Rasch Measurement Theory is that the analyses are sample‐free and item‐free (Bond et al., 2020). We generated Rasch person measures for each participant at both timepoints for the CNC 8 items and CNC 10 items and used these data for responsiveness analyses.

We calculated the pooled standard deviation (SD_pooled_), effect size, and the standardized response mean using MedCalc version 17.6. We interpreted the effect size and standardized response mean at 0.20, 0.50, and 0.80 as a small, moderate, and large responsiveness (Husted et al., 2000). The standard error of measurement (SEM) was calculated using the following formula SEM = SD_pooled_($\sqrt{\left(1-r\right)}$, where *r* reflected the Wright's Person Separation Reliability value. We used the SD_pooled_ to calculate the distribution‐based MCIDs at 0.20 (small), 0.33 (minimally important), and 0.50 (medium) standard deviations. The 0.20 SD MCID reflects a small but important group‐level change, 0.33 SD MCID reflects a minimally important change relevant for clinical interpretation, and a 0.50 SD MCID reflects a medium sized effect (Crosby et al., 2003; Eton et al., 2004). We calculated the minimal detectable change using a 95% confidence interval (MDC_95_ = 1.96 × SEM × $\sqrt{2}$). For both the CNC 8‐item and CNC 10‐item versions, we determined whether the difference between the baseline and follow‐up mean person measures exceeded the distribution‐based MCID. For both the CNC 8‐item and CNC 10‐item versions, we determined the proportion of participants that exceeded the MDC_95_ after two consecutive weeks of rehabilitation. To exceed the MDC_95_, a participant's change score could indicate either a decline or improvement in neurobehavioral function that was beyond measurement error.

## 2 RESULTS

### 2.1 Participants

Participants (*n* = 40) were a mean (SD) age of 35 (12.7) years, mostly males (*n* = 35; 88%), with traumatic brain injuries (*n* = 36; 90%). Participants from the Post‐Acute Care study were enrolled within the first 90 days postinjury while the Familiar Auditory Sensory Training and repetitive transcranial magnetic stimulation participants were enrolled between 90 days and 2 years after injury. Most participants were within 90 days of their injury date (19; 47%), with 11 participants between 91 and 180 days and 10 participants were more than 180 days from their injury date at time of CNC administration. At study enrollment, most participants had a tracheostomy (29; 73%) and there was an even distribution of participants in the vegetative state/unresponsive wakefulness syndrome (16; 40%) and minimally conscious state (21; 53%).

### 2.2 Indices of reliability and responsiveness

The CNC 8‐item ordinal total raw scores ranged from 0 to 32 and were transformed to equal‐interval Rasch person measures, ranging from −3.81 to 4.58 logits. The CNC 10‐item ordinal total raw scores ranged from 0 to 40 and were transformed to equal‐interval Rasch person measures ranging from −4.18 to 4.59 logits. We provide a transformation table for converting ordinal total raw scores to equal‐interval Rasch person measures in logits for both CNC 8‐item and 10‐item versions (Table 1). The Wright's Person Separation Reliability indices for the CNC 8 items and CNC 10 items, remained at 0.87 and 0.89, respectively (Table 2).

**TABLE 1:** Total raw score to Rasch person measure conversion for the 8‐item and 10‐item Coma Near Coma Scale from least to most neurobehavioral function.

	CNC total raw score	CNC‐8 item person measure	CNC‐10 item person measure
Less neurobehavioral function More neurobehavioral function	40	–	−4.18
	38	–	–2.97
	36	–	–2.25
	34	–	–1.81
	32	–3.81	–1.48
	30	–2.59	–1.21
	28	–1.86	–0.96
	26	–1.40	–0.74
	24	–1.04	–0.53
	22	–0.74	–0.33
	20	–0.46	–0.14
	18	–0.21	0.06
	16	0.04	0.27
	14	0.29	0.48
	12	0.55	0.72
	10	0.83	0.99
	8	1.17	1.30
	6	1.58	1.70
	4	2.16	2.25
	2	3.12	3.17
	0	4.58	4.59

**TABLE 2:** Indices of Responsiveness for the Coma Near Coma Scale With and Without Painful Test Stimuli.

Coma Near‐Coma version	Baseline mean (SD)	Follow‐up mean (SD)	SD_pooled_	Wright's Person Separation Reliability	Standard error of measurement	Effect size (using SD_pooled_)	Lower 95% CI	Upper 95% CI	Standardized response mean
8 items (without pain items)	–0.80 (1.4)	–0.43 (1.1)	1.25	0.87	0.45	0.29	–0.03	0.61	0.29
10 items (with pain items)	–0.72 (1.2)	–0.43 (1.0)	1.12	0.89	0.37	0.26	–0.12	–0.57	0.24

Abbreviations: CI, confidence interval; SD, standard deviation; SD_pooled_, pooled standard deviation of baseline and follow‐up measures.

Notation: Data calculated using Rasch person measures (logits), a higher number indicates more neurobehavioral function.

The small (0.20) effect sizes and standardized response means produced similar values (0.22–0.25 logits) and indicate that sample size computations, for each version, would not differ when designing clinical trials (Table 3). The small (0.20 SD), minimally important (0.33 SD), and medium (0.50 SD) distribution‐based MCIDs are almost equivalent for the Coma Near‐Coma‐8 item and CNC 10‐item versions (Table 3). The CNC 8 items has consistently larger distribution‐based MCIDs than the CNC 10 items (Table 3). The mean difference between the baseline and follow‐up scores after receiving an intervention (Table 2) was 0.37 and 0.35 for the CNC 8 items and CNC 10 items, respectively, which exceeds the respective small (0.20 SD) distribution‐based MCIDs (Table 3).

**TABLE 3:** Minimal detectable change and distribution‐based minimal clinically important differences for the Coma Near‐Coma Scale with and without painful test stimuli.

Coma Near‐Coma Version	MDC_95_	MCID 0.20 SD	MCID 0.33 SD	MCID 0.50 SD
8 items (without pain items)	1.25	0.25	0.41	0.63
10 items (with pain items)	1.03	0.22	0.37	0.56

Abbreviations: MCID, minimal clinically important difference; MDC_95_, minimal detectable change using 95% confidence intervals.

The MDC_95_ findings indicate the CNC 10‐item version has less measurement error yet both versions identified similar proportions of persons making neurobehavioral change beyond measurement error (Table 3) . Specifically, the CNC 8‐item version identified 12 (30%) participants and the CNC 10‐item version identified 13 (33%) participants as making a change beyond measurement error or a “true” change. Of these patients making “true” change, the CNC 8‐item version identified 9/12 (75%) participants as improved and 3 as declined (25%); the CNC 10‐item version identified 10/13 (77%) participants as improved and 3 as declined (23%).

## 3 DISCUSSION

The preliminary evidence reported here, addresses the identified need for additional research on the measurement properties of the CNC scale, including responsiveness (Seel et al., 2010). The reported findings suggest that the CNC 8 items (without painful test stimuli) and CNC 10 items (with painful stimuli) have comparable reliability and precision for detecting change in neurobehavioral function. As postulated, the Wright's Person Separation Reliability values did not change with the addition of 8 participants. The finding that the shorter CNC 8 items has comparable precision for detecting change to the longer CNC 10‐item supports previous findings suggesting that pain is a distinct concept from neurobehavioral function (Weaver et al., 2020). The Wright sample‐independent person separation reliability indices for both CNC versions remain below the 0.90 threshold for making consistently reliable individual patient treatment decisions.

The minor differences in person separation reliability and MDC_95_ between the CNC 10 items and CNC 8 items indicate, that both versions identified comparable proportions of participants making a change beyond measurement error. Participants were identified as improvers if the person measure increased and were identified as decliners if the person measure decreased at the second CNC assessment. It is important to include decliners when identifying participants who made a change using the MDC_95_ because it illuminates the need for additional clinical reasoning to determine whether there is something unobservable at the bedside hindering progress (i.e., medication changes, subclinical seizures, or hydrocephalus).

The distribution‐based MCID values are sample‐dependent because the equation uses the group's SD_pooled_ (Altman & Bland, 2005). The standard error of measurement and MDC_95_ values, are less sample‐dependent because the equation utilizes Wright's Person Separation Reliability coefficient in addition to SD_pooled_. The CNC 8 items and CNC 10 items had comparable reliability coefficients. The CNC 8 items had greater distribution‐based MCIDs, standard error of measurement, and MDC_95_ values because of a larger SD_pooled_ compared to the CNC 10‐item version. The MDC_95_ was greater than the distribution‐based MCID values for both CNC versions indicating that if the distribution‐based MCIDs are utilized it is possible an improvement or decline is not “true” but within the range of measurement error. This evidence suggests that the distribution‐based MCID values should be used to interpret group‐level changes and the MDC_95_ should be used to interpret change at the individual patient level.

The standard error of measurement and MDC_95_ can be used clinically. When a patient has a score of −1.86 logits (raw score: 28) on the CNC 8‐item version, the standard error of measurement indicates that the patient's range of ability is −2.31 to −1.41 logits (total measure in logits ± standard error of measurement). The patient's progress can be considered “true change” beyond measurement error (MDC_95_) when the same patient achieves a total measure of −0.61 logits or greater (raw score: 20 or lower; a difference of 8 or more). This information can support clinicians to determine whether an applied intervention is supporting the patient's neurobehavioral recovery.

Our study addresses the need for indices of responsiveness for neurobehavioral function assessments used for patients with disorders of consciousness (Seel et al., 2010; Weaver et al., 2020). This preliminary study supports the clinical and research utility of the CNC 8 items for measuring change in neurobehavioral function with reduced administration burden. Our study further suggests no additional value for determining change in neurobehavioral function is achieved by inflicting pain on patients through administration of the pain items. Indices of responsiveness, such as the CNC 8‐item MDC_95_, can support clinicians to make data‐driven decisions about patient recovery whereas indices such as the CNC 8‐item distribution‐based MCID and effect size can inform clinical trials examining treatment effectiveness at the group level.

### 3.1 Study limitations

The indices of responsiveness we report should be interpreted cautiously because they were generated from a small sample of adults with disorders of consciousness. Future studies should substantiate our findings using larger samples. For patients with disorders of consciousness, establishing an anchor‐based MCID reflecting family, care partner, or clinician perception of change is an important area of future inquiry.

## 4 CONCLUSION

Our preliminary evidence supports the clinical and research utility of the CNC 8 items, as it demonstrates adequate responsiveness to neurobehavioral change without administering the two pain items. When evaluating change at the group level the CNC 8‐item effect size and distribution‐based MCIDs should be used. When evaluating change for an individual patient, the CNC 8‐item MDC_95_ should be used to determine when observed change is beyond measurement error.

### PEER REVIEW

The peer review history for this article is available at https://publons.com/publon/10.1002/brb3.3120.

## Data Availability

Deidentified data from this study are available upon request.

## References

[brb33120-bib-0001] Altman, D. G. , & Bland, J. M. (2005). Standard deviations and standard errors. Bmj, 331(7521), 903. 10.1136/bmj.331.7521.903 16223828 PMC1255808

[brb33120-bib-0002] Bond, T. G. , Yan, Z. , & Heene, M. (2020). Applying the Rasch model fundamental measurement in the human sciences (4th edn.). Routledge.

[brb33120-bib-0003] Crosby, R. D. , Kolotkin, R. L. , & Williams, G. R (2003). Defining clinically meaningful change in health‐related quality of life. Journal of Clinical Epidemiology, 56(5), 395–407. 10.1016/s0895-4356(03)00044-1 12812812

[brb33120-bib-0004] Eton, D. T. , Cella, D. , Yost, K. J. , Yount, S. E. , Peterman, A. H. , Neuberg, D. S. , Sledge, G. W. , & Wood, W. C. (2004). A combination of distribution‐ and anchor‐based approaches determined minimally important differences (MIDs) for four endpoints in a breast cancer scale. Journal of Clinical Epidemiology, 57(9), 898–910. 10.1016/j.jclinepi.2004.01.012 15504633

[brb33120-bib-0005] Giacino, J. T. , Ashwal, S. , Childs, N. , Cranford, R. , Jennett, B. , Katz, D. I. , Kelly, J. P. , Rosenberg, J. H. , Whyte, J. , Zafonte, R. D. , & Zasler, N. D. (2002). The minimally conscious state: Definition and diagnostic criteria. Neurology, 58(3), 349–353. 10.1212/wnl.58.3.349 11839831

[brb33120-bib-0006] Giacino, J. T. , Whyte, J. , Nakase‐Richardson, R. , Katz, D. I. , Arciniegas, D. B. , Blum, S. , Day, K. , Greenwald, B. D. , Hammond, F. M. , Pape, T. B. , Rosenbaum, A. , Seel, R. T. , Weintraub, A. , Yablon, S. , Zafonte, R. D. , & Zasler, N. (2020). Minimum competency recommendations for programs that provide rehabilitation services for persons with disorders of consciousness: A position statement of the American Congress of Rehabilitation Medicine and the National Institute on Disability, Independent Living and Rehabilitation Research Traumatic Brain Injury Model Systems. Archives of Physical Medicine and Rehabilitation, 101(6), 1072–1089. 10.1016/j.apmr.2020.01.013 32087109

[brb33120-bib-0007] Guyatt, G. H. , Osoba, D. , Wu, A. W. , Wyrwich, K. W. , & Norman, G. R. (2002). Methods to explain the clinical significance of health status measures. Mayo Clinic Proceedings, 77(4), 371–383. Retrieved from 10.4065/77.4.371 11936935

[brb33120-bib-0008] Husted, J. A. , Cook, R. J. , Farewell, V. T. , & Gladman, D. D. (2000). Original articles: Methods for assessing responsiveness. A critical review and recommendations. Journal of Clinical Epidemiology, 53, 459–468. 10.1016/S0895-4356(99)00206-1 10812317

[brb33120-bib-0009] Kerlinger, F. N. , & Lee, H. B. (2000). Foundations of behavioral research (4th edn.). Thompson Learning, Inc.

[brb33120-bib-0010] Laureys, S. , Faymonville, M. E. , Peigneux, P. , Damas, P. , Lambermont, B. , Del Fiore, G. , Degueldre, C. , Aerts, J. , Luxen, A. , Franck, G. , Lamy, M. , Moonen, G. , & Maquet, P. (2002). Cortical processing of noxious somatosensory stimuli in the persistent vegetative state. Neuroimage, 17(2), 732–741. 10.1006/nimg.2002.1236 12377148

[brb33120-bib-0011] Mallinson, T. , Pape, T. L.‐B. , & Guernon, A. (2016). Responsiveness, minimal detectable change, and minimally clinically important differences for the Disorders of Consciousness Scale. Journal of Head Trauma Rehabilitation, 31(4), E43–E51. 10.1097/HTR.0000000000000184 26360003

[brb33120-bib-0012] Pape, T. (2015). rTMS: A treatment to restore function after severe TBI. Retrieved from https://clinicaltrials.gov/ct2/show/study/NCT02366754

[brb33120-bib-0013] Pape, T. L.‐B. , Mallinson, T. , & Guernon, A. (2014). Psychometric properties of the disorders of consciousness scale. Archives of Physical Medicine and Rehabilitation, 95(9), 1672–1684. 10.1016/j.apmr.2014.04.015 24814459

[brb33120-bib-0014] Pape, T. L.‐B. , Rosenow, J. M. , Steiner, M. , Parrish, T. , Guernon, A. , Harton, B. , Patil, V. , Bhaumik, D. K. , Mcnamee, S. , Walker, M. , Froehlich, K. , Burress, C. , Odle, C. , Wang, X. , Herrold, A. A. , Zhao, W. , Reda, D. , Mallinson, T. , Conneely, M. , & Nemeth, A. J. (2015). Placebo‐controlled trial of familiar auditory sensory training for acute severe traumatic brain injury: A preliminary report. Neurorehabilitation and Neural Repair, 29(6), 537–547. 10.1177/1545968314554626 25613986

[brb33120-bib-0015] Rappaport, M. (2000). The Coma/Near Coma Scale. Retrieved from https://www.tbims.org/combi/cnc

[brb33120-bib-0016] Rappaport, M. (2005). The Disability Rating and Coma/Near‐Coma scales in evaluating severe head injury. Neuropsychol Rehabil, 15(3‐4), 442–453. 10.1080/09602010443000335 16350985

[brb33120-bib-0017] Rappaport, M. , Dougherty, A. M. , & Kelting, D. L. (1992). Evaluation of coma and vegetative states. Archives of Physical Medicine and Rehabilitation, 73(7), 628–634. Retrieved from https://proxy.library.georgetown.edu/login?url= https://ovidsp.ovid.com/ovidweb.cgi?T=JS&CSC=Y&NEWS=N&PAGE=fulltext&D=emed5&AN=22247909 1622317

[brb33120-bib-0018] Schnakers, C. , Chatelle, C. , Vanhaudenhuyse, A. , Majerus, S. , Ledoux, D. , Boly, M. , Bruno, M.‐A. , Boveroux, P. , Demertzi, A. , Moonen, G. , & Laureys, S. (2010). The Nociception Coma Scale: A new tool to assess nociception in disorders of consciousness. Pain, 148(2), 215–219. 10.1016/j.pain.2009.09.028 19854576

[brb33120-bib-0019] Seel, R. T. , Sherer, M. , Whyte, J. , Katz, D. I. , Giacino, J. T. , Rosenbaum, A. M. , Hammond, F. M. , Kalmar, K. , Pape, T. L.‐B. , Zafonte, R. , Biester, R. C. , Kaelin, D. , Kean, J. , & Zasler, N. (2010). Assessment scales for disorders of consciousness: Evidence‐based recommendations for clinical practice and research. Archives of Physical Medicine and Rehabilitation, 91(12), 1795–1813. 10.1016/j.apmr.2010.07.218 21112421

[brb33120-bib-0020] Van De Winckel, A. , Kozlowski, A. J. , Johnston, M. V. , Weaver, J. , Grampurohit, N. , Terhorst, L. , Juengst, S. , Ehrlich‐Jones, L. , Heinemann, A. W. , Melvin, J. , Sood, P. , & Mallinson, T. (2022). Reporting Guideline for RULER: Rasch Reporting Guideline for rehabilitation research—Explanation and elaboration. Archives of Physical Medicine and Rehabilitation, 103(7), 1487–1498. 10.1016/j.apmr.2022.03.019 35436496

[brb33120-bib-0021] Weaver, J. A. , Cogan, A. M. , O'brien, K. A. , Hansen, P. , Giacino, J. T. , Whyte, J. , Bender Pape, T. , Van Der Wees, P. , & Mallinson, T. (2022). Determining the hierarchy of Coma Recovery Scale‐Revised Rating Scale Categories and alignment with aspen consensus criteria for patients with brain injury: A Rasch analysis. Journal of Neurotrauma, 39, 1417–1428. 10.1089/neu.2022.0095 35570725 PMC9529298

[brb33120-bib-0022] Weaver, J. A. , Liu, J. , Guernon, A. , Pape, T. B. , & Mallinson, T. (2020). Psychometric properties of the Coma Near‐Coma Scale for adults in disordered states of consciousness: A Rasch analysis. Archives of Physical Medicine and Rehabilitation, 102, 591–597. 10.1016/j.apmr.2020.10.119 33161008

[brb33120-bib-0023] Weaver, J. A. , Liu, J. , Guernon, A. , Pape, T. B. , & Mallinson, T. (2021). Psychometric properties of the coma Near‐Coma Scale for adults in disordered states of consciousness: A Rasch analysis. Archives of Physical Medicine and Rehabilitation, 102(4), 591–597. 10.1016/j.apmr.2020.10.119 33161008

[brb33120-bib-0024] Williams, M. W. , & Smith, E. L. (2017). Clinical utility and psychometric properties of the Disability Rating Scale with individuals with traumatic brain injury. Rehabilitation Psychology, 62(3), 407–408. 10.1037/rep0000168 28836811

[brb33120-bib-0025] Zasler, N. D. , Formisano, R. , & Aloisi, M. (2022). Pain in persons with disorders of consciousness. Brain Sciences, 12(3), 300. https://www.mdpi.com/2076‐3425/12/3/300 35326257 10.3390/brainsci12030300 PMC8946117

[brb33120-bib-0026] Zilliox, M. J. , Foecking, E. M. , Kuffel, G. R. , Conneely, M. , Saban, K. L. , Herrold, A. A. , Kletzel, S. L. , Radke, J. R. , Walsh, E. , Guernon, A. , Pape, A. , Ripley, D. L. , Patil, V. , Pacheco, M. S. , Rosenow, J. M. , Bhaumik, R. , Bhaumik, D. , & Pape, T. L. B (2022). An initial miRNA profile of persons with persisting neurobehavioral impairments and states of disordered consciousness after severe traumatic brain injury. Journal of Head Trauma Rehabilitation, Publish Ahead of Print, 10.1097/htr.0000000000000821 36350037

